# Personality traits and perceived cognitive function in lebanese healthcare professionals

**DOI:** 10.1186/s40359-023-01139-w

**Published:** 2023-03-31

**Authors:** Sara Moussa, Iris Achkouty, Diana Malaeb, Anthony Ghosn, Sahar Obeid, Souheil Hallit

**Affiliations:** 1 Faculty of Medicine, University of Balamand, Koura, Lebanon; 2 School of Medicine and Medical Sciences, Holy Spirit University of Kaslik, P.O. Box 446, Jounieh, Lebanon; 3 College of Pharmacy, Gulf Medical University, Ajman, UAE; 4 School of Pharmacy, Lebanese International University, Beirut, Lebanon; 5 Social and Education Sciences Department, School of Arts and Sciences, Lebanese American University, Jbeil, Lebanon; 6 Applied Science Research Center, Applied Science Private University, Amman, Jordan; 7 Research Department, Psychiatric Hospital of the Cross, Jal Eddib, Lebanon

**Keywords:** Personality traits, Cognitive function, Healthcare Professionals, Extroversion, Conscientiousness, Negative emotionality

## Abstract

**Background:**

The healthcare field, a well-known field associated with variety of stressors, leaves healthcare professionals at an increased risk of both physical and mental problems. COVID-19 pandemic has recently been added to the stressful factors by endangering further the cognitive function of healthcare workers. On another hand, personality traits have been shown to have pervasive associations with functioning across various cognitive domains. Thus, this study aims to evaluate association between personality traits and perceived cognitive function among healthcare professionals in Lebanon during the collapsing period (following the severe economic crisis and the COVID-19 pandemic).

**Methods:**

This cross-sectional study was conducted between November 2021 and January 2022 enrolled 406 Lebanese participants using the convenience sampling technique for data collection. Healthcare professionals from all specialties who received the online link to the survey were eligible to participate. The Big Five Inventory-2 (BFI-2) and Fact Cog scale were used to assess personality traits and cognitive function.

**Results:**

After adjustment over all variables (age, gender, household crowding index, physical activity index, marital status, profession and the other four personality traits), higher negative emotionality was significantly associated with a worse cognitive function, whereas more extroversion and conscientiousness were significantly associated with a better cognitive function.

**Conclusion:**

Our study adds to the narrow body of research revolving around the relationship between personality traits and perceived cognitive function in Lebanese healthcare professionals during these hard times in Lebanon. These results show that the choice of these cognitive processes is strongly affected by different personality traits, such as extroversion, conscientiousness, and negative emotionality. This study encourages the need to conduct further research that assess the changes in cognition in life stressors along with personality traits.

## Background

Cognition refers to a set of skills that is required to achieve several tasks such as accessing services, processing and understanding information, expressing oneself, recalling spoken instructions, using technology, critically weighing options, and making the right decisions [1]. Cognitive impairment is a disruption in any of these skills. Several approaches from clinical neuropsychology tried to come up with an exact definition. They concluded that a performance deficit, such as one standard deviation below the mean on one or more areas of cognitive function, would define cognitive impairment [2–4]. Various domains such as executive and motor function, information processing, spatial skill, verbal and visual memory fall under the umbrella of cognitive function [5]. It has been shown that in some countries, such as the UK, work-related cognitive function decline is common affecting around 1.1 million people who suffer from conditions that are caused by or made worse by work [6]. The healthcare field is known for all the stressors that accompany it. In fact, the work of doctors and surgeons can give rise to both physical and mental problems [6, 7]. Nevertheless, they are more prone to occupational stress, that increases when their responses to the workload are overwhelming [8]. In a recent survey, 38% of surgeons though that work had negative effects on their health, and 79.1% of doctors identified stress or the overall workload, to be important factors [9]. One is tempted to think that surgeons are, by nature, less affected by work stressors and can cope better where in fact, surgery often is both emotionally and physically demanding, therefore a surgeon’s psychological health should not be ignored or overlooked [6, 8].

Personality traits have been found to have extensive correlations with cognitive functioning across several cognitive domains [10]. Researchers discovered the Big 5 personality traits, which were the most common, after studying numerous personalities from different genders and nations. These include openness, conscientiousness, extroversion, agreeableness, and negative emotionality [11]. John & Srivastava describe openness as being intellectual, polished, and independent-minded; and conscientiousness as being responsible, orderly, and dependable [12]. On the other hand, agreeableness refers to the tendency to be altruistic, trusting, modest, and compliant [13], whereas negative emotionality refers to the tendency to be emotionally unstable, and to experience negative emotions such as anger, anxiety, and depression [14]. Extrovert people attend to the outside world, exchanging energy there by interacting with people and the environment. They are sociable and prefer to communicate, learn and engage by talking, and taking the initiative in work and in relationships [15]. On the other hand, introvert people shift their focus on their inner world of ideas and experiences. They tend to prefer communication and learning through writing and reflection [16]. Some authors investigate the association between these personality traits and cognitive function. They found that extroversion, for example, may be related to better cognitive function because it engenders sensitivity to external reward, which may promote diligence [17]. Furthermore, extroversion is also linked to increased levels of positive affect which seems to be strongly influencing cognitive function [18].

Persons who choose to work in the healthcare field tend to be high in conscientiousness. They tend to be dutiful, self-disciplined, organized, ambitious, hardworking, persistent, efficient at carrying out tasks, and achievement orientated [19]. Some people confuse it with being socially responsible [20]. Recent work has demonstrated that conscientiousness is the best personality predictor of job performance, and academic achievements, and that it has tremendous effects on cognitive ability [21]. On one hand, conscientiousness and cognitive function are related to many of the same outcome variables such as job performance, training performance, and delinquency, whereas on the other hand, some examinations have suggested very little relationship between these variables [22]. Therefore, further research needs to be conducted in an attempt to resolve some of this conflict. As previously discussed, negative emotionality is associated with negative emotions such as anxiety, fear, and anger. There is a well-established relationship between negative emotionality and cognitive decline, particularly among the elderly. Previous data suggest that 15–25% of elderly persons experience high levels of negative emotionality [23]. Thus, negative emotionality mostly is related to poorer performance on a number of cognitive measures, [24]. Several cross-sectional and longitudinal studies showed that individuals high in negative emotionality are at increased risk for developing mild cognitive impairment [25].

Lebanon is a tiny nation on the Mediterranean Sea’s eastern shore. Its first case of COVID-19 was discovered on February 21, 2020 [27], in the midst of the country’s economic collapse. All Lebanese residents, particularly healthcare employees, were severely impacted by this economic crisis [28]. Then there was the explosion at the Beirut Port, which made half of the capital’s hospitals inoperable, injured a large number of healthcare personnel, and presented new obstacles to the health care system. To put it differently, healthcare professionals have been challenged by the resulting weak healthcare system, scarcity of resources, low salaries, increasing workload [29, 30] and previous traumas as they try to combat a pandemic, severely affecting their cognitive function [31]. Between June and July 2020, a cross-sectional survey was done among healthcare employees at a tertiary hospital in Lebanon. It revealed that nearly two-thirds of them think their job is placing them at greater danger and causing them to be more stressed. Furthermore, 59% were more concerned about contracting COVID19 [32]. This can be attributed to the high infectivity and mortality rates, long incubation period of the virus, and all the ambiguity about transmission and required precautions [33], along with the ongoing worry about the wellbeing of family members and close contacts [34].

Bearing all that in mind, Lebanon’s economic crisis and financial collapse have placed healthcare workers in a challenging situation with minimal psychological support. Thus, immediate attention to this field is essential to rebuilding trust and strengthening the healthcare system. On another hand, little to no research has investigated the relationship between personality traits and subjective cognitive function in healthcare professionals during such times. Up until today, studies tackling this link have either focused only on one profession, did not target the various areas of the country, or have been conducted in a different time frame, which is a crucial element in this study. Therefore, this study aimed to assess the association between personality traits and perceived cognitive function among a sample of Lebanese healthcare professionals in a collapsing country.

## Methods 

### Participants

This cross-sectional study was conducted between November 2021 and January 2022. A total of 406 participants (200 females, 206 males) was recruited through convenience sampling through several areas in Lebanon’s governorates. Participants were healthcare professionals from all specialties who received an online link to the survey. They were encouraged to visit the link which would guide them to the consent form, purpose of the study, anonymity, and the questionnaire. There were no fees for participating in the study. Eligible individuals who gave consent to respond willingly participated whereas those who refused to complete the survey were excluded. All methods were performed in accordance with the relevant guidelines and regulations.

### Minimal sample size calculation

According to the G-power software, a minimum of 316 healthcare professionals was deemed necessary to have enough statistical power, based on a 5% risk of error, 80% power, f^2^ = 2.5% and 10 factors to be entered in the multivariable analysis.

### Questionnaire

The questionnaire was in Arabic and required approximately 10 to 15 min to complete. The questionnaire assessed demographic and health characteristics of participants, including age, gender, healthcare field, and the number of persons in the household and the number of rooms in the house, excluding the bathroom and the kitchen, to calculate the household crowding index (the number of rooms divided by the number of persons) [35]. Physical activity index was calculated by multiplying the frequency by duration by strength of the physical activity in general [36]. The second part consisted of the following scales:

### The big five inventory-2 (BFI-2)

This 60-item scale describes personality in terms of five main domains, each of 12 items,: extroversion, agreeableness, conscientiousness, negative emotionality and open mindedness [37]. Each domain is assessed with certain facets: Extroversion (with facets of Sociability, Assertiveness, and Energy Level), Agreeableness (Compassion, Respectfulness, and Trust), Conscientiousness (Organization, Productiveness, and Responsibility), Negative Emotionality (Anxiety, Depression, and Emotional Volatility), and Open-Mindedness (Intellectual Curiosity, Aesthetic Sensitivity, and Creative Imagination) [37]. Each item, of the total 60 items, is scored on a basic numeric scoring of 1 (disagree) to 5 (agree). The Arabic version was provided by Dr Soto. The Cronbach’s alpha values were as follows: extroversion (0.89), agreeableness (0.91), conscientiousness (0.89), negative emotionality (0.90) and open mindedness (0.90).

### Fact cog scale

This scale has 37 items encompassing four aspects. The first aspect ispatients’ perceived cognitive impairments (CogPCI), under which 20 items fall, and is mainly centered around how one perceives his/her cognitive impairments; i.e. “I have had trouble concentrating”. The second aspect isperceived cognitive abilities (CogPCA), under which 9 items fall, and addresses how one perceives his/her cognitive abilities, whether one is able or not, i.e. “I have been able to concentrate”. The third aspect are deficits observed or commented on by others (CogOth), which is exemplified in 4 items centered around what others have told you, i.e. “Other people have told me I seem confused”. The last aspect which also has 4 items is theimpact of cognitive changes on quality of life (CogQOL), which tackles how the problems interfere with daily living, i.e. “These problems have interfered with the quality of my life” [35]. Respondents indicate the frequency of each occurrence over the last 7 days on a 5-point scale. In CogPCI and CogOth, 0 refers to never, and 4 refers to several times per day, whereas in the CogPCA and CogQOL subscales, 0 corresponds to not at all and 4 to very much. In our study, The FACT-Cog TOTAL score was used, which can be obtained by summing the individual subscale scores. It ranges from 0 to 148, with higher scores indicating better cognitive functioning. The Cronbach’s alpha for the total scale was 0.95. The Arabic version was provided by the FACIT organization. The scale is usually used in cancer patients but the FACIT organization allowed us to use it in the general population as well.

### Statistical analysis

Data analysis was conducted using the 25th version of the SPSS software. The Cronbach’s alpha values were calculated for all scales and subscales. The normality of distribution of the cognitive function score was confirmed via a calculation of the skewness and kurtosis; values for asymmetry and kurtosis between − 2 and + 2 are considered acceptable to prove normal univariate distribution [38]. To conduct the bivariate analysis, the Student t and ANOVA tests were used to compare two and three means respectively. Pearson correlations were assessed between cognitive function and personality traits. For the multivariable analysis, partial correlations were evaluated between cognitive function and each personality trait, while controlling for all variables (age, gender, household crowding index, physical activity index, marital status, profession and the other four personality traits). Partial correlations were chosen as regression models for multiple purposes. First, Pearson and partial correlation can be compared in an easier way, as the two of them align between − 1 to + 1. Second, regression coefficients and partial correlations both generate the exact inferential results because they have equal p-values. In psychological analysis, correlations of 0.1, 0.2 and 0.3 were considered as having a small, medium and large effect sizes, respectively [39]. Significance is considered with a p-value < 0.05.

## Results

Married participants had a significantly higher mean cognitive function score compared to single ones (Table 1). Higher household crowding index (r=-0.14; p = 0.004) was significantly associated with lower cognitive function, whereas higher physical activity index (r = 0.18; p < 0.001) was significantly associated with higher cognitive function. Age (r = 0.09; p = 0.071) and financial burden (r = 0.08; p = 0.132) were not significantly associated with cognitive function.

**Table 1:** Bivariate analysis of categorical factors associated with the cognitive function score (N = 406)

Variable	Mean ± SD	*p*	Effect size
**Gender**		0.984	0.002
Male (N = 206)	94.57 ± 17.60		
Female (N = 200)	94.61 ± 19.20		
**Marital status**			
Single / widowed / divorced (N = 224)	91.63 ± 18.08	**< 0.001**	0.365
Married (N = 182)	98.24 ± 18.14		
**Profession**		0.952	0.052
Physician (N = 102)	94.11 ± 16.22		
Pharmacist (N = 55)	94.76 ± 16.11		
Dentist (N = 24)	92.46 ± 15.19		
Nurse (N = 78)	96.26 ± 20.99		
Physical therapist (N = 37)	93.68 ± 25.68		
Other (N = 110)	64.55 ± 17.38		

Numbers in bold indicate significant p-values.

### Zero order and partial correlations between personality traits and cognitive function

After adjustment over all variables (age, gender, household crowding index, physical activity index, marital status, profession and the other four personality traits), higher negative emotionality was significantly associated with a worse cognitive function, whereas more extroversion and conscientiousness were significantly associated with a better cognitive function (Table 2).

**Table 2:** Zero order and partial correlations between personality traits and cognitive function (N = 406)

	Zero order	Partial correlation
Extroversion	**r = 0.38**	**r = 0.15**
Agreeableness	**r = 0.26**	r= -0.24
Conscientiousness	**r = 0.35**	**r = 0.17**
Negative emotionality	**r= -0.34**	**r= -0.23**
Open mindedness	**r = 0.28**	r = 0.03

Zero order corresponds to correlations between variables without any adjustment over other variables. Partial corresponds to correlations adjusted for age, gender, household crowding index, physical activity index, marital status, profession and the other four personality traits. Values in bold correspond to statistically significant correlations (p < 0.05).

## Discussion

This study demonstrated that more negative emotionality was significantly associated with worse perceived cognitive function, whereas more extroversion, and conscientiousness were significantly associated with better perceived cognitive function.

Conscientiousness is characterized by the relatively stable pattern in the tendencies to follow certain social norms and rules, to be goal-directed, to be planful, and to delay gratification [40]. This personality trait enables individuals to better weigh the pros and cons of a given situation, and potentially have a better cognitive function [41]. The positive relationship that we found between conscientiousness and perceived cognitive functioning solidified previous findings [10, 42–46]. In fact, conscientiousness was associated with better performance across all five cognitive domains: memory, speed-attention-executive, visuospatial ability, fluency, and numeric reasoning [10]. Persons higher in conscientiousness demonstrate a slower rate of cognitive decline over an assigned period of time [42]. This was further proved when measuring cognitive impairment in Alzheimer patients, where conscientiousness was associated with a decreased incidence of cognitive impairment and slowed the pace of cognitive decline [43]. Furthermore, some individuals with low cognitive ability even showed that they developed an increased conscientiousness in compensation to their attenuated cognitive function [44]. Not only that, but when Sutin et al. investigated distinct facets of conscientiousness, such as industriousness and responsibility, they found that the latter significantly contributed to a better cognitive performance and a healthier cognitive aging [45]. Finally, some authors observed employees at work who had low levels of conscientiousness and came up with the concept of “cognitive failure”. This failure was manifested in the increased risk of accidents and unsafe work behaviors when conscientiousness is low [46]. Thus, solidifying evidence exist for the link between conscientiousness and perceived cognitive function.

In our study, extroversion is significantly associated with better perceived cognitive function. This positive association was supported by previous authors [46–49] and our present study. Previous data demonstrates the effects of extroversion in influencing both mood and performance in specific cognitive tasks [49]. This personality trait was associated with better overall cognitive function and even showed domain-specific associations, such as speed-attention-executive and fluency [46]. When examined on the long run-in age related cognitive decline, old people with higher levels of extroversion and social engagement showed slower cognitive decline [48]. When Chapman et al. compared extroversion to introversion, moderate extraversion was associated with lower risk of cognitive impairment in both case-control and co-twin designs [47]. Given that, authors agreed that higher extroversion could potentially act as a defense mechanism against stress-related deterioration of cognitive functions, which is a crucial finding in the current pandemic [48].

The interplay between negative emotionality and cognitive function has always been an intriguing topic. In this study, we found that more negative emotionality was significantly associated with worse perceived cognitive function corroborating the findings of previous papers [50–55]. This is not surprising since the mechanisms of emotion and cognition are intertwined from early perception to reasoning [50]. The type of emotional response generates, influences, and shapes the cognitive processes that an individual experiences [51]. For example, cognitive functioning improves less after eliciting negative emotions as compared to positive emotions [52]. A possible reason is that negative emotions negatively influence specific cognitive processes, such as those involved in memory and judgment [53]. On the other hand, different types of elicited emotions have different effects. For instance, information with positive content is learned better than negative information, reflecting the negative relationship between negative emotions and cognitive functioning [54]. Furthermore, a study conducted on preschool children revealed that positive emotions generated an overall improvement in children’s cognitive functioning as compared to negative emotions, which led to a decline in cognitive functioning [55].

### Clinical implications

Our study adds to the narrow body of research revolving around the relationship between personality traits and perceived cognitive function in Lebanese healthcare professionals during the current pandemic. This helps better analyze the cognitive processes, which professionals use when facing life stressors in the healthcare field. These results show that the choice of these cognitive processes is strongly affected by different personality traits, such as extroversion, conscientiousness, and negative emotionality. Also, this gives insight for the development of valuable tools to improve students’ interpersonal skills. Furthermore, it will aid in developing strategies that will be implemented by these healthcare workers, by observing these variables closely in their patients and giving more value to the correlates mentioned that have a major effect on the treatment plans. Understanding the impact of the COVID-19 pandemic and the daily stressors that healthcare professionals are subject to in Lebanon would help establish more preventative guidelines and mental health awareness campaigns.

### Limitations

All studies have limitations; our study is no exception to that. Its cross-sectional nature gives it a lower internal validity in comparison with experimental studies. Also, it limits the ability to draw causal conclusions. The two scales used are not validated in Lebanon in their English versions. The questionnaire was self-administered, which poses a risk for information bias considering the risk of misunderstanding the questions by either under or over-estimating them by the participants. Information bias can also be because self-reported measures were employed in the present research where participants self-reported themselves on personality types and cognitive function. The sample might have also been representative of a certain healthcare field more than the other, which also poses a risk of bias. The scales used were not validated in the country. Thus, further studies taking all these limitations into consideration, are required to better assess the associations in this study. Although the sample is relatively small, the association between perceived cognition and personality traits is worthwhile regardless of situation or population. It is noteworthy to mention that the results were similar to those found in the literature. This congruence supports the generalizability of these associations regardless of the situation or population.

## Conclusion

The results showed a positive relationship between extroversion, conscientiousness with perceived cognitive function, whereas negative emotionality was significantly associated with worse perceived cognitive function. To our knowledge, no previous research has looked into a direct link between these variables in Lebanon especially in these times. Our results demonstrate the pivotal role that personality traits might play in important life outcomes, highlight the need to more routinely incorporate measures to protect the cognitive functioning of healthcare workers especially during such hard times, and encourage further research about the changes in cognition, especially perceived or subjective cognition, in times of major life stressors and the processes by which personality traits influence these changes.

## Data Availability

The datasets generated and/or analyzed during the current study are not publicly available due to the authors do not have the right to share any data information as per their institutions’ policies but are available from the corresponding author on reasonable request.
